# Power estimation using simulations for air pollution time-series studies

**DOI:** 10.1186/1476-069X-11-68

**Published:** 2012-09-20

**Authors:** Andrea Winquist, Mitchel Klein, Paige Tolbert, Stefanie Ebelt Sarnat

**Affiliations:** 1 Department of Environmental Health, Rollins School of Public Health, Emory University, 1518 Clifton Road, NE, Atlanta, GA 30322, USA

**Keywords:** Statistical power, Time-series studies, Air pollution epidemiology

## Abstract

**Background:**

Estimation of power to assess associations of interest can be challenging for time-series studies of the acute health effects of air pollution because there are two dimensions of sample size (time-series length and daily outcome counts), and because these studies often use generalized linear models to control for complex patterns of covariation between pollutants and time trends, meteorology and possibly other pollutants. In general, statistical software packages for power estimation rely on simplifying assumptions that may not adequately capture this complexity. Here we examine the impact of various factors affecting power using simulations, with comparison of power estimates obtained from simulations with those obtained using statistical software.

**Methods:**

Power was estimated for various analyses within a time-series study of air pollution and emergency department visits using simulations for specified scenarios. Mean daily emergency department visit counts, model parameter value estimates and daily values for air pollution and meteorological variables from actual data (8/1/98 to 7/31/99 in Atlanta) were used to generate simulated daily outcome counts with specified temporal associations with air pollutants and randomly generated error based on a Poisson distribution. Power was estimated by conducting analyses of the association between simulated daily outcome counts and air pollution in 2000 data sets for each scenario. Power estimates from simulations and statistical software (G*Power and PASS) were compared.

**Results:**

In the simulation results, increasing time-series length and average daily outcome counts both increased power to a similar extent. Our results also illustrate the low power that can result from using outcomes with low daily counts or short time series, and the reduction in power that can accompany use of multipollutant models. Power estimates obtained using standard statistical software were very similar to those from the simulations when properly implemented; implementation, however, was not straightforward.

**Conclusions:**

These analyses demonstrate the similar impact on power of increasing time-series length versus increasing daily outcome counts, which has not previously been reported. Implementation of power software for these studies is discussed and guidance is provided.

## Background

In a given study, the power of a particular analysis is the probability of identifying a statistically significant association if a non-random association truly exists. Given a specified type 1 error probability, power depends on several factors including study design, the distribution of the outcome and type of analytical model, sample size, the strength of the effect of interest, the distribution of the exposure, and covariation between exposure and its covariates [1,2]. When planning a study, researchers need to ensure that the study can be expected to have adequate power for the questions of interest.

Ensuring sufficient power can be a challenge in time-series studies of the acute health effects of air pollution. The complex relationships between pollutants and the many other factors (e.g., temporal trends and meteorology) impacting the acute health outcomes of interest in these studies lead to the need for complex modeling for control of confounding and assessment of interactions [3]. In these models, there is often a high degree of covariation among pollutants, and between pollutants and other model variables [4,5], which typically diminishes effect estimate precision and decreases power to identify air pollutant effects [6]. In addition, air pollution health effects over short time intervals are often small; lower effect sizes also decrease power. A characteristic of these studies that distinguishes them from many other types of studies with regard to power is that there are two dimensions of sample size (time-series length and the magnitude of the daily outcome counts) which both impact power, but not necessarily in the same way. For an extreme illustration, compare a 5000-day time series with a mean of 2 events per day to a 2-day time series with a mean of 5000 events per day. Both studies have a total of 10,000 events but they are allocated over a different length of time. While most studies have less extreme study design options than these, the relative impact of the two aspects of sample size on study power is not necessarily apparent and has implications for study design decisions.

Estimating power when designing time-series studies of the acute health effects of air pollution can also be particularly challenging. While methods for sample size calculation for studies using multivariate generalized linear models have been developed [2,7,8] and statistical software packages are available for estimating power for such studies (ex. G*Power [1,9] (which is publicly available at no cost), and PASS [10,11]), these calculations generally rely on simplifying assumptions that may not be valid in a given study, and require specification of parameters that may be difficult to estimate based on available information.

Here we use simulations to estimate power for specific analyses within an air pollution time-series study using observed data from Atlanta, Georgia, and illustrate the impact of various study design factors on study power. Of particular interest was comparing the relative impact of the two dimensions of sample size on power for analyses in these studies. We also compare the power estimates obtained using simulations with those obtained using statistical software, using the simulations as the gold standard.

## Methods

### General approach

Power was estimated for a time-series study of acute air pollution health effects. Data on daily emergency department (ED) visit counts from 18 hospitals and daily values for air pollution and meteorological variables for the 8/1/98 to 7/31/99 time period in Atlanta [12] were used to generate simulated data sets with specified temporal associations between daily outcome counts and air pollutants. Power calculations were conducted for scenarios chosen to represent a range of pollutant-outcome combinations of interest, various magnitudes of effect, and various time-series lengths and mean daily outcome counts.

### Observed data

The pollutants considered in the scenarios included a commonly examined air pollutant (24-h average fine particulate matter, PM_2.5_), a less commonly considered pollutant with sporadic spikes in levels (24-h average total water-soluble PM_2.5_ metals), and pollutants that are correlated over time [carbon monoxide (CO, daily 1-h maximum) and elemental carbon (EC, 24-h average)]. The distributions of daily measurements of these pollutants in the observed Atlanta data are described in Table 1. The ED visit outcomes for which data were simulated were cardiovascular outcomes of common interest in time-series studies of air pollution health effects, and included a cardiovascular disease grouping (CVD, ICD-9 codes 402,410-414,427,428,433-437,440,443,444,451-453), dysrhythmia (ICD-9 code 427), and cardiac arrest (CA, ICD-9 code 427.5). The average daily counts for these outcomes in the observed data were 42.9 for CVD, 10.7 for dysrhythmia, and 3.1 for CA.

**Table 1:** Overall average pollutant levels for pollutants considered in power simulations, Atlanta, 8/1/98-7/31/99

**Pollutant**	**Mean**	**Standard Deviation**	**25**^**th**^**Percentile**	**Median**	**75**^**th**^**Percentile**	**Maximum**	Skewness*	**Number of days missing for moving average of lags 0-2**
PM_2.5_ (μg/m³), 24-h average	19.42	9.35	12.50	17.54	24.76	53.24	1.00	31
Total water- soluble PM_2.5_ metals (μg/m³), 24-h average	0.03	0.03	0.01	0.02	0.04	0.20	2.07	58
Elemental carbon (EC) (μg/m³), 24-h average	2.26	1.74	1.26	1.88	2.60	15.61	2.83	22
Carbon monoxide (CO) (ppmV), daily 1-h maximum	1.47	1.30	0.60	0.98	1.81	9.42	2.27	44

*Skewness was calculated using SAS version 9.2 with the formula: “ $\frac{n}{\left(n-1\right)\left(n-2\right)}\sum_{i=1}^{n}w_{i}^{3/2}{\left(\frac{x_{i}-{\bar{x}}_{w}}{s_{w}}\right)}^{3}$where *n* is the number of non-missing values for a variable, $x_{i}$ is the *i*th value of the variable, ${\bar{x}}_{w}$ is the sample average, *s* is the sample standard deviation, and *w*_*i*_=1 for all *i*=1,…,*n*“ [25].

### Scenarios

In Scenario Set 1, we examined the relative impact on power of increasing or decreasing the number of ED visits per day (e.g., to reflect the impact of altering the number of hospitals providing ED visit data for the study, or conducting the study in a location with a larger or smaller population) and increasing the length of the time series. For this scenario set, we simulated daily counts for the CVD, dysrhythmia, and CA outcomes in relation to PM_2.5_, with the risk ratio (RR) being based on analysis of the observed data (CVD: RR = 1.024 per 10 μg/m³, dysrhythmia: RR = 1.026 per 10 μg/m³, CA: RR = 1.104 per 10 μg/m³). Calculated predicted daily counts were multiplied by 0.5, 1, 2, 3, 4, or 5 (to represent various degrees of reduction or expansion of the number of hospitals or the population in the study); and time-series lengths of 1, 2, 3 and 4 years were considered.

In Scenario Set 2, we examined the impact on power of varying the true underlying RR. For this scenario set, we simulated daily counts for the CVD and dysrhythmia outcomes in relation to total water-soluble PM_2.5_ metals. Total water-soluble PM_2.5_ metals serves as an example of a pollutant for which there is little prior information about the expected RR. We selected RR estimates of 1.03, 1.05 and 1.07 (per standard deviation increase in water soluble metals- 0.03 μg/m³) for this scenario set (reflecting preliminary results for the association between total water-soluble PM_2.5_ metals and dysrhythmia, and uncertainty about the true RR). The time-series length was one year. Calculated predicted daily counts were doubled to more closely reflect the mean daily counts after a planned expansion of the number of hospitals providing ED data for the Atlanta study.

In Scenario Set 3, we compared the power for analyses that did or did not control for covarying pollutants. For this scenario set we simulated data for the CVD and dysrhythmia outcomes in relation to both CO and EC. Both of these pollutants were found to be associated with these outcomes in single-pollutant models in prior analyses [12] and in current analyses of our observed data when using a data set with the same days with missing values for EC and CO (CVD-CO: RR per 1 ppmV = 1.039, p = 0.0007; CVD-EC: RR per 2 μg/m³ = 1.052, p = 0.0088; dysrhythmia-CO: RR per 1 ppmV = 1.067, p = 0.0037; dysrhythmia-EC: RR per 2 μg/m³ = 1.106, p = 0.0097). Since CO and EC are correlated (Spearman correlation coefficient = 0.6), it was of interest to assess possible confounding of each pollutant’s effect by the other pollutant in two-pollutant models. In two-pollutant models using the observed data, the risk ratios for CVD were 1.032 per 1 ppmV for CO (p = 0.0207) and 1.022 per 2 μg/m³ for EC (p = 0.3432), and the risk ratios for dysrhythmia were 1.049 per 1 ppmV for CO (p = 0.0774) and 1.058 per 2 μg/m³ for EC (p = 0.2265). We compared power estimates for each pollutant for analyses using single pollutant and two-pollutant models, using the risk ratios from the single and two-pollutant models for the observed data. Calculated predicted daily counts were again doubled, and time-series lengths of 1, 2, 3, and 4 years were considered.

### Epidemiologic models

The power analyses were designed for Poisson generalized linear models that allowed for overdispersion and controlled for temperature [cubic spline, with knots at the 25^th^ and 75^th^ percentiles (12.22°C and 23.89°C), for the moving average (lags 0–2) of the daily average temperature], dew point [cubic spline, with knots at the 25^th^ and 75^th^ percentiles (4.33°C and 18.28°C), for the moving average (lags 0–2) of daily average dew point], day of week and periods of hospital participation (indicator variables), and underlying time trends (cubic spline for time with seasonal knots for four seasons). After accounting for time trends, it was determined that it was not necessary to account for autocorrelation in the outcome data. Pollutants were included in the models as the moving average of lags 0–2, and risk ratios were calculated per approximate standard deviation increase in pollutant levels.

### Power calculations using simulations

Simulated daily outcome counts were generated for each scenario with average daily counts corresponding to those in the one year of observed data; with the specified temporal associations with air pollutants; and with associations with variables relating to time and meteorology (and other pollutants in two-pollutant models) that reflected the associations in the one year of observed data. First, predicted mean daily outcome counts (expected counts) were calculated for each day, using models as specified above, as a function of daily observed values for the variables in the model and the estimated or *a priori* parameter value for each variable. The parameter values for pollutants were either specified *a priori* (in Scenario Set 2) or estimated from the observed data using models as specified above (in Scenario Sets 1 and 3). Parameter values for other variables in the model (time and meteorology variables) were estimated from the observed data using models as specified above, except that models for generation of parameter estimates for Scenario Set 2 (which had *a priori* specification of pollutant risk ratios) did not include the pollution variable. The calculated daily expected counts were scaled to have the same mean as in our observed data for Atlanta during 8/1/98-7/31/99. For some scenarios, these expected counts were multiplied by factors of 0.5, 1, 2, 3, 4, or 5 to reflect the potential impact of increasing or decreasing the number of hospitals reporting ED data for the study or the population size. For scenarios estimating the effect of using time series more than 1 year in length, the observed daily values for all variables in the model (including variables for time trends), and the expected daily outcome counts were repeated for subsequent years. Once data sets with appropriately scaled expected daily outcome counts and the appropriate time-series length had been generated, 2000 data sets were created for each scenario. Simulated daily outcome counts in these data sets were generated based on a Poisson distribution with the daily mean being the scaled daily expected outcome counts. Analytic models, as specified above, were then run on each of the 2000 simulated data sets for each scenario. Power was calculated as the percentage of the data sets for each scenario that showed a statistically significant association between the pollutant and the simulated daily outcome counts, using a significance level of 0.05. All simulations and analyses of simulated data were conducted using SAS version 9.2 (SAS Institute, Inc., Cary, North Carolina, USA).

### Power calculations using statistical software

Power was also estimated for each scenario using the algorithms for estimation of power for multivariate Poisson generalized linear models in G*Power [1,9] and PASS [10,11] software. For G*Power, estimates using the enumeration procedure of Lyles, *et al.*[7] are reported. The algorithms in these software packages require specification of the base rate (“Exp(β0)”), the risk ratio, the proportion of the variance of the pollution variable explained by other variables in the model (“R-squared other X”), the distribution of the predictor variable (“distribution of X1”), the sample size, the mean exposure period, alpha, and the number of tails [1,9,11].

Both software packages ask the user to supply the “base rate” (Exp(β0)). The G*Power instructions explain Exp(β0) as “the mean event rate assumed under H_0_.” [9] The PASS instructions explain Exp(β0) as, “the response rate that occurs when all covariates are equal to zero.” [11] Interpretation of these instructions is not straightforward. Since the intercept (β0) depends on the coding of the variables in the model, it is not clear what to enter for Exp(β0). It is theoretically possible to estimate the expected daily rate under the null hypothesis of no effect of air pollution based on the observed risk ratio, observed mean daily counts, and pollutant levels. However, in our data, this estimation procedure yielded counts differing little from the observed mean daily counts because risk ratios are close to the null (data not shown). We considered two other methods for calculating Exp(β0), including exponentiation of the intercept from the models and use of the observed mean daily count. Each method produced different power estimates. Ultimately, we chose the observed mean daily count as our best estimate of Exp(β0), because it led to power estimates that were closest to those from the simulations. The risk ratio was as specified in each scenario.

“R-squared other X” was obtained from linear regression models that regressed the air pollution variable on the other variables in the analytic model. Due to the limited number of options available for the distribution of X1, a normal distribution was assumed for all pollutants, with the mean and variance estimated from the observed data. The sample size was the number of days in the time series in each scenario (accounting for the number of days with missing pollutant values in the actual data set for comparability with simulations), and the mean exposure period was 1 day. All calculations were two-tailed with alpha = 0.05.

## Results

**Figure 1:**
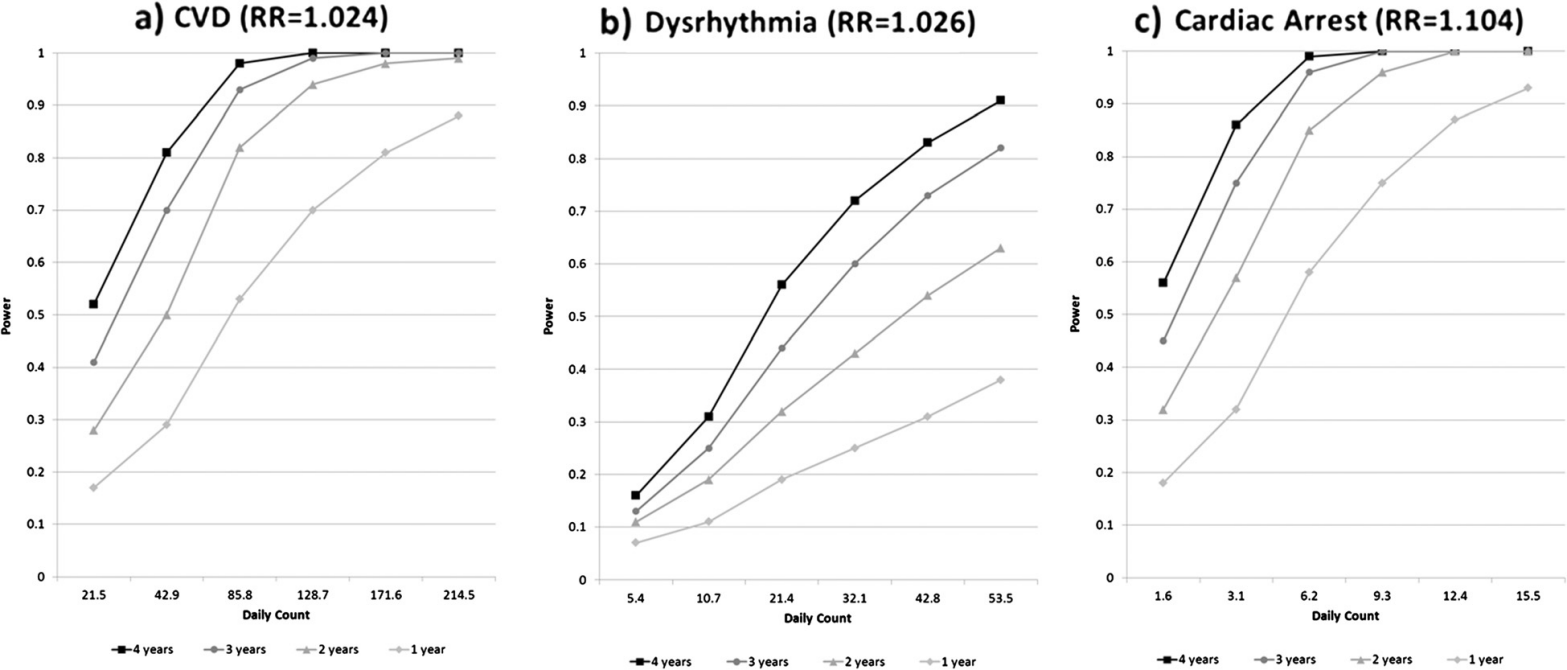
**Power estimates from simulations varying time-series length and daily outcome counts (Scenario Set 1).** Power estimates are for PM2.5 in relation to CVD (**a**), dysrhythmia (**b**), and cardiac arrest (**c**). Simulated daily outcome counts were generated for each scenario with average daily counts corresponding to average counts in one year of observed data; with the specified temporal associations with air pollutants; and with associations with variables relating to time and meteorology that reflected the associations in the one year of observed data. For each scenario, the mean daily counts were scaled appropriately, time series of the specified length were created, and 2000 simulated data sets were generated based on a Poisson distribution. Power was calculated as the percentage of the 2000 simulated data sets for each scenario that showed a statistically significant association between the pollutant and the simulated daily outcome counts, using a significance level of 0.05.

**Figure 2:**
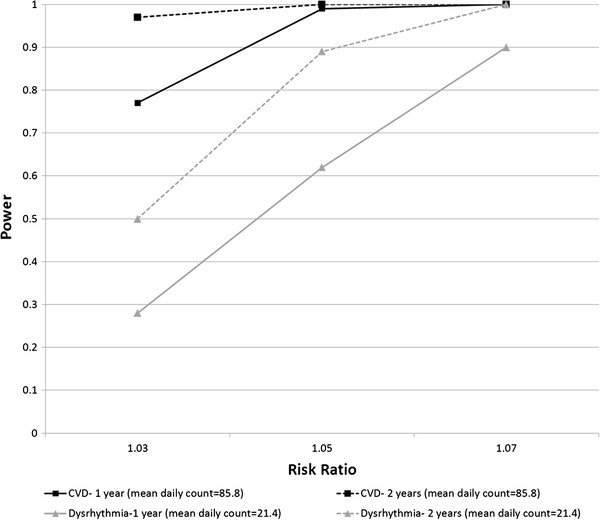
**Power estimates from simulations varying risk ratio and time-series length (Scenario Set 2).** Power estimates are for total water soluble PM_2.5_ metals in relation to CVD and dysrhythmia. Mean daily outcome counts were held constant at twice the mean daily counts in the observed data. Simulated daily outcome counts were generated for each scenario with average daily counts corresponding to average counts in one year of observed data; with the specified temporal associations with air pollutants; and with associations with variables relating to time and meteorology that reflected the associations in the one year of observed data. For each scenario, the mean daily counts were scaled appropriately, time series of the specified length were created, and 2000 simulated data sets were generated based on a Poisson distribution. Power was calculated as the percentage of the 2000 simulated data sets for each scenario that showed a statistically significant association between the pollutant and the simulated daily outcome counts, using a significance level of 0.05.

**Figure 3:**
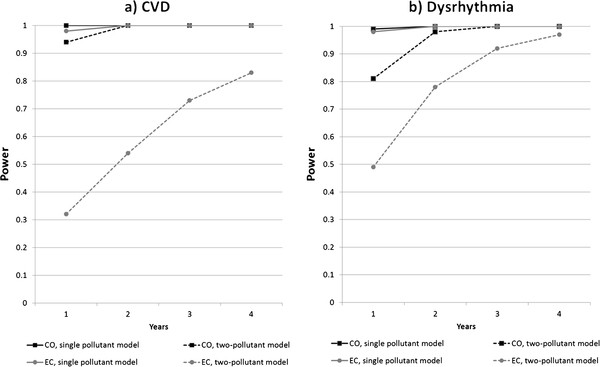
**Power estimates from simulations comparing single pollutant models and two-pollutant models (Scenario Set 3).** Power estimates are for elemental carbon (EC) and carbon monoxide (CO) in relation to CVD and dysrhythmia, with varying time-series length. Mean daily outcome counts were held constant at twice the mean daily counts in the observed data. Simulated daily outcome counts were generated for each scenario with average daily counts corresponding to average counts in one year of observed data; with the specified temporal associations with air pollutants; and with associations with variables relating to time and meteorology and the other pollutant in the two-pollutant models that reflected the associations in the one year of observed data. For each scenario, the mean daily counts were scaled appropriately, time series of the specified length were created, and 2000 simulated data sets were generated based on a Poisson distribution. Power was calculated as the percentage of the 2000 simulated data sets for each scenario that showed a statistically significant association between the pollutant and the simulated daily outcome counts, using a significance level of 0.05.

The power estimates from the simulations for the various scenarios are shown in Table 2 and Figures 1, 2 and 3. In scenario set 1, increasing the time-series length and increasing the average number of visits per day both increased power, with both having a similar impact on power (Figure 1). For example for the CVD outcome, compared with the scenario with a time-series length of 1 year and the original, unamplified mean daily visits (mean daily count = 42.9, power = 0.29), the scenario that tripled the time-series length but kept the original mean daily counts increased power to 0.70, and the scenario that tripled the mean daily counts but kept the time-series length at 1 year increased power to 0.70 as well**.** In scenario set 1, power was estimated to be very low (e.g. <0.31 for dysrhythmia) when using outcomes with very low daily counts (such as dysrhythmia with a mean of 5.4-10.7 visits per day, or cardiac arrest with a mean of 1.6-3.1 visits per day), even when using time series as long as 4 years for dysrhythmia. Power was somewhat better with low counts for cardiac arrest than for dysrhythmia at similar mean daily counts due to the high risk ratio for the cardiac arrest-PM_2.5_ association in our data set.

**Table 2:** Power estimates for time-series models of air pollution under various scenarios

	**Outcome**	**Pollutant(s) in model**	**Specified Risk Ratio**	**Series Length**	**Mean daily visits**	**Power from simulations**	G*Power Estimates†
Scenario Set 1	CVD	PM_2.5_	1.024 per 10 μg/m³	1 year	21.5	**0.17**	0.16
					42.9	**0.29**	0.28
					85.8	**0.53**	0.49
					128.7	**0.70**	0.66
					171.6	**0.81**	0.78
					214.5	**0.88**	0.86
				2 years	21.5	**0.28**	0.28
					42.9	**0.50**	0.49
					85.8	**0.82**	0.78
					128.7	**0.94**	0.92
					171.6	**0.98**	0.97
					214.5	**0.99**	0.99
				3 years	21.5	**0.41**	0.39
					42.9	**0.70**	0.66
					85.8	**0.93**	0.92
					128.7	**0.99**	0.98
					171.6	**1.00**	1.00
					214.5	**1.00**	1.00
				4 years	21.5	**0.52**	0.50
					42.9	**0.81**	0.79
					85.8	**0.98**	0.97
					128.7	**1.00**	1.00
					171.6	**1.00**	1.00
					214.5	**1.00**	1.00
	Dysrhythmia	PM_2.5_	1.026 per 10 μg/m³	1 year	5.4	**0.07**	0.08
					10.7	**0.11**	0.11
					21.4	**0.19**	0.18
					32.1	**0.25**	0.25
					42.8	**0.31**	0.31
					53.5	**0.38**	0.38
				2 years	5.4	**0.11**	0.12
					10.7	**0.19**	0.18
					21.4	**0.32**	0.32
					32.1	**0.43**	0.45
					42.8	**0.54**	0.56
					53.5	**0.63**	0.65
				3 years	5.4	**0.13**	0.15
					10.7	**0.25**	0.25
					21.4	**0.44**	0.45
					32.1	**0.60**	0.61
					42.8	**0.73**	0.73
					53.5	**0.82**	0.82
				4 years	5.4	**0.16**	0.19
					10.7	**0.31**	0.32
					21.4	**0.56**	0.56
					32.1	**0.72**	0.73
					42.8	**0.83**	0.85
					53.5	**0.91**	0.92
	Cardiac Arrest	PM_2.5_	1.104 per 10 μg/m³	1 year	1.6	**0.18**	0.22
					3.1	**0.32**	0.38
					6.2	**0.58**	0.64
					9.3	**0.75**	0.81
					12.4	**0.87**	0.91
					15.5	**0.93**	0.96
				2 years	1.6	**0.32**	0.39
					3.1	**0.57**	0.65
					6.2	**0.85**	0.91
					9.3	**0.96**	0.98
					12.4	**1.00**	1.00
					15.5	**1.00**	1.00
				3 years	1.6	**0.45**	0.54
					3.1	**0.75**	0.82
					6.2	**0.96**	0.98
					9.3	**1.00**	1.00
					12.4	**1.00**	1.00
					15.5	**1.00**	1.00
				4 years	1.6	**0.56**	0.67
					3.1	**0.86**	0.91
					6.2	**0.99**	1.00
					9.3	**1.00**	1.00
					12.4	**1.00**	1.00
					15.5	**1.00**	1.00
Scenario Set 2	CVD	Total water- soluble PM_2.5_ metals	1.03 per 0.03 μg/m³	1 year	85.8	**0.77**	0.72
			1.05 per 0.03 μg/m³			**0.99**	0.99
			1.07 per 0.03 μg/m³			**1.00**	1.00
			1.03 per 0.03 μg/m³	2 years		**0.97**	0.95
			1.05 per 0.03 μg/m³			**1.00**	1.00
			1.07 per 0.03 μg/m³			**1.00**	1.00
	Dysrhythmia		1.03 per 0.03 μg/m³	1 year	21.4	**0.28**	0.24
			1.05 per 0.03 μg/m³			**0.62**	0.55
			1.07 per 0.03 μg/m³			**0.90**	0.83
			1.03 per 0.03 μg/m³	2 years		**0.50**	0.44
			1.05 per 0.03 μg/m³			**0.89**	0.85
			1.07 per 0.03 μg/m³			**1.00**	0.99
Scenario Set 3	CVD	Elemental carbon (EC) (single pollutant model)§	EC: 1.052 per 2 μg/m³	1 year	85.8	**EC: 0.98**	0.99
			EC: 1.052 per 2 μg/m³	2 years		**EC: 1.00**	1.00
			EC: 1.052 per 2 μg/m³	3 years		**EC: 1.00**	1.00
			EC: 1.052 per 2 μg/m³	4 years		**EC: 1.00**	1.00
		Carbon monoxide (CO) (single pollutant model)§	CO: 1.039 per 1 ppmV	1 year		**CO: 1.00**	1.00
			CO: 1.039 per 1 ppmV	2 years		**CO: 1.00**	1.00
			CO: 1.039 per 1 ppmV	3 years		**CO: 1.00**	1.00
			CO: 1.039 per 1 ppmV	4 years		**CO: 1.00**	1.00
		Elemental carbon (EC) and Carbon monoxide (CO) in two- pollutant model	EC: 1.022 per 2 μg/m³	1 year		**EC: 0.32**	0.35
			CO: 1.032 per 1 ppmV			**CO: 0.94**	0.95
			EC: 1.022 per 2 μg/m³	2 years		**EC: 0.54**	0.61
			CO: 1.032 per 1 ppmV			**CO: 1.00**	1.00
			EC: 1.022 per 2 μg/m³	3 years		**EC: 0.73**	0.78
			CO: 1.032 per 1 ppmV			**CO: 1.00**	1.00
			EC: 1.022 per 2 μg/m³	4 years		**EC: 0.83**	0.89
			CO: 1.032 per 1 ppmV			**CO: 1.00**	1.00
	Dysrhythmia	Elemental carbon (EC) (single pollutant model)	EC: 1.106 per 2 μg/m³	1 year	21.4	**EC: 0.98**	0.99
			EC: 1.106 per 2 μg/m³	2 years		**EC: 1.00**	1.00
			EC: 1.106 per 2 μg/m³	3 years		**EC: 1.00**	1.00
			EC: 1.106 per 2 μg/m³	4 years		**EC: 1.00**	1.00
		Carbon monoxide (CO) (single pollutant model)	CO: 1.067 per 1 ppmV	1 year		**CO: 0.99**	0.99
			CO: 1.067 per 1 ppmV	2 years		**CO: 1.00**	1.00
			CO: 1.067 per 1 ppmV	3 years		**CO: 1.00**	1.00
			CO: 1.067 per 1 ppmV	4 years		**CO: 1.00**	1.00
		Elemental carbon (EC) and Carbon monoxide (CO) in two- pollutant model	EC: 1.058 per 2 μg/m³	1 year		**EC: 0.49**	0.55
			CO: 1.049 per 1 ppmV			**CO: 0.81**	0.79
			EC: 1.058 per 2 μg/m³	2 years		**EC: 0.78**	0.84
			CO: 1.049 per 1 ppmV			**CO: 0.98**	0.97
			EC: 1.058 per 2 μg/m³	3 years		**EC: 0.92**	0.95
			CO: 1.049 per 1 ppmV			**CO: 1.00**	1.00
			EC: 1.058 per 2 μg/m³	4 years		**EC: 0.97**	0.99
			CO: 1.049 per 1 ppmV			**CO: 1.00**	1.00

All models controlled for temperature (cubic splines with knots at 12.22°C and 23.89°C, for the moving average of daily average temperature (lags 0–2 days)), dew point (cubic splines with knots at 4.33°C and 18.28°C, for the moving average of daily average dew point (lags 0–2 days)), weekday, hospital participation periods, and underlying time trends (cubic spline for time with seasonal knots for four seasons).

† G*Power estimates were generated using the Lyles Enumeration Procedure. Power estimates obtained using PASS were very similar (all within 2 percentage points of the G* Power results) and are not shown. G*Power estimates use the mean daily count as expβ0 and account for days with missing pollutant values.

§Single pollutant models have missing values on the same days as the two-pollutant models.

In scenario set 2, as expected, for each outcome, power increased as the specified risk ratio increased (Figure 2). The power also differed by outcome, largely due to differences in mean daily counts.

In scenario set 3, power for both EC and CO was substantially lower in the two-pollutant models than in the single pollutant models (Figure 3), with the power consistently greater for CO than for EC. The difference in power between the pollutants in the two-pollutant models decreased with increasing time-series length.

Power estimates obtained using G*Power software are also shown in Table 2. Results obtained using PASS software were nearly identical to results obtained using G*Power software (always within 2 percentage points) and are not shown. Power estimates from statistical software were very similar to estimates from the simulations when using the mean daily count as Exp(β0) and accounting for the number of days with missing pollutant values. If the number of days with missing pollutant values was not accounted for, power was over estimated (data not shown). In addition, if a more straightforward interpretation of Exp(β0) was used, in which the exponentiated intercept from the model was used as Exp(β0), power was substantially overestimated in some scenarios and substantially underestimated in other scenarios (data not shown).

## Discussion

These analyses illustrate the impact of increasing time-series length and mean daily counts on power for air pollution time-series studies, and the way in which that impact varies for different pollutants, magnitudes of the risk ratio, and daily outcome counts. Moreover, they illustrate the potential usefulness of simulations in estimating power for such studies, as well as a reliable method for using statistical software for estimating power for such studies.

Scenario Set 1 illustrates the important point that studies considering outcomes with very low mean daily counts (<10 per day) will have very low power, even with time series up to 4 years in length, when the risk ratio is low. Scenario Set 1 also illustrates the two dimensions of sample size in this type of study: the time-series length and the mean daily counts. To our knowledge, the relative impact of these two dimensions of sample size on power in time-series studies of air pollution health effects has not previously been directly examined. Our simulation results suggest that the power can be increased by increasing either the mean daily count (e.g., by increasing the number of hospitals contributing data to the study) or the time-series length, with both having a similar impact on the expected value of power if the joint distribution of the outcome, pollution levels, and covariates is fixed. In our simulations, by repeating the values of all covariates in the model in years after the first year, the distribution of the pollution variable and the covariance between pollution and other variables in the model were held constant to allow a ‘pure’ comparison of the effect of the two dimensions of sample size. However, in a real-world study, changing the length of the time series would likely change the observed distribution of pollution levels and covariates in a way that changing the mean daily counts would not. This could lead to changes in the actual power of a study that are not solely due to the change in the time series length. As an example, consider the hypothetical example of a 2-day time series with a mean daily count of 5,000 events per day. This study could have excellent power to detect an air pollution effect if the two days had very different air pollution levels or very poor power if the two days had similar pollution levels. That is, there is an element of chance in the days selected. By contrast, a 5000-day time series with a mean of 2 events per day could have a more representative distribution of pollution levels and covariates and would also have the potential for evaluation of dose–response curves, seasonal effects, and interactions in a way that the 2-day time series would not.

In practice increasing each of these dimensions of sample size may have challenges. The length of the time series may be limited by the availability of historical data or the time available for prospective data collection. The mean daily outcome counts may be limited by the population size in the study area, and increasing the number of hospitals may increase exposure measurement error if the geographic area is substantially expanded. The scenarios considered here reflect those considered in published air pollution time-series studies, which have varied widely in terms of the length of the time series and the average number of daily events. Some studies have had long time series and a high average number of daily events as a result of conducting studies in large metropolitan areas or of combining data from multiple cities [13-15]. For example, Strickland *et al.*[14] examined a 12-year time series with daily measurements of air pollutants and a mean of 18.9 pediatric asthma visits per day during the warm season and 22.8 per day during the cold season. Le Tertre *et al.*[15] analyzed data for 8 European cities with city-specific time series varying in length from 3 to 7 years and a mean number of daily hospital admissions for cardiac conditions in each city ranging from 16 to 138. However, other studies have considered shorter time periods and/or lower average number of daily events [16-18]. Some have had short time series due to an interest in studying the impact of short-term events such as wildfires (e.g., a study by Delfino *et al.*[16] with a 46-day time series). Some studies have had a low mean number of daily events due to a small population size but local concerns about the health effects of air pollution (e.g., a study by Ulirsch *et al.*[17] that combined counts of several different types of health care visits to achieve a 5.5-year time series with mean daily event counts of 18.0-21.1 for respiratory outcomes and 2.0-4.9 for cardiovascular outcomes), or an interest in locally collected data with a level of covariate detail not usually available in large administrative data sets [e.g., a study by Stieb *et al.*[18] that examined a 3.75-year time series of ED visits, with information on smoking status, the presenting complaint, and date of symptom onset, but with low mean daily ED visit counts (e.g., 10.9 for the ‘all respiratory’ outcome group, and <4 for cardiovascular case groups)]. In some studies, mean daily counts are moderate for some outcomes but very low for other outcomes (e.g., a study by Slaughter *et al.*[19] that examined a 6-year time series for hospital admissions and a 6.5-year time series for ED visits in which the mean daily event counts were 12.2 for ‘all respiratory’ ED visits, but <10 for all other outcome groups including mean counts of ≤3 for some outcome groups). Our findings can help guide investigators when considering power in studies such as these, with very short time series or very low average daily event counts.

Scenario Set 2 illustrates how power depends on the specified effect size. The impact of the effect size on power can also be seen in Scenario Set 1, in the surprisingly high power seen for cardiac arrest in spite of low daily counts, which was due to the high risk ratio for the cardiac arrest-PM_2.5_ association observed in our data set. However, it should be noted that effect estimates for outcomes with low daily counts can be very unstable. Use of an effect estimate for an outcome with low daily counts from a short time series in a power calculation may give misleading power estimates.

Scenario Set 3 compares the power for analyses that do or do not control for covarying pollutants. Power to detect a statistically significant effect may be reduced in multi-pollutant models for several reasons. First, the risk ratios for the pollutants are often smaller in the multi-pollutant model than in the single pollutant models due to the control for positive confounding between the pollutants. Second, collinearity between pollutants can cause model instability and inflate parameter estimate variances in multipollutant models [6], with an accompanying decrease in power. Finally, if pollutants have missing observations on different days, the multi-pollutant model will have more missing values than the single pollutant models, leading to increased parameter estimate variances and reduced power [20]. In our scenarios, the single pollutant models were made to have the same number of missing days as the two-pollutant models; therefore, this was not the reason for the difference in power. In scenario set 3, although the RR was greater for EC than for CO in the two-pollutant model for dysrhythmia, the power was greater for CO in all models. The reason for this is that in addition to the odds ratio, the proportion of the variance of a pollutant that is explained by other variables in the model and the variability of pollutant values also affect power. In the two pollutant models, the proportion of the variance explained by other variables in the model was higher for EC than for CO, and the coefficient of variation was lower for EC than for CO. Both of these factors will decrease power for EC. These findings demonstrate the important fact that the power to detect an effect may not be the same for all pollutants in a multipollutant model, due to different pollutant distributions and different relationships with other variables in the model. All else being equal, pollutants that have high correlations with other variables in the model or that have low variability will have lower power.

When the power estimates from the simulations were compared with those obtained from statistical software, we found that the power estimated by the statistical software was very similar to that estimated through the simulations. The similarity between power estimates from simulations and power software was observed despite the challenges in calculating some of the parameters required for the statistical software. For example, several of the pollutants had skewed distributions, which could have led to inaccuracies in resulting power estimates due to the assumption of a normal pollutant distribution and to use linear regression models for estimation of the proportion of the pollution variable variance that was due to other model variables. In addition, we found that implementation of the software for time-series studies was not straightforward due to difficulties involved in defining Exp(β0). Our results show that using the mean daily count in power calculations for these types of studies (with risk ratios that are close to the null) may be a reasonable approach to estimating Exp(β0). The more straightforward approach based on a simple interpretation of the instructions, of exponentiating β0 from the models, led to inaccuracies in the power estimates. When using power software to estimate power, it is also important to account for the expected degree of missing data, as was done here. Power estimates from software that did not account for missing data overestimated power.

Power in time-series analyses could also vary in ways not considered in these analyses. There are many ways to model a particular pollutant-outcome relationship with regard to pollutant characterization, lag structure, control for confounding due to time trends and meteorological factors, and type of analysis [21]. The question, “What is the power?” can only be answered for a specific pollutant-outcome model with specified effects and a specified modeling strategy. Model specifications can affect power by impacting the covariation between the pollutant of interest and other variables in the model. Power can also be affected by factors that may not be directly controllable in the study design phase, such as missing values [20] and measurement error, which can decrease power [22,23].

The magnitudes of the power estimates in these simulations are specific to our model specifications, as well as to the pollutant, outcome and covariate distributions and observed risk ratios in our observed data set. However, the conclusions relating to how various factors impact power are generalizable to different model specifications within the framework of Poisson generalized linear models. They are also generalizable to different outcomes, different pollutants and different locations. In any scenario, the same factors influence power in the same way.

While power calculations such as these can be helpful in assuring that a study is well-designed, they must be interpreted correctly. While increasing the sample size will increase power, this should not be interpreted to mean that an estimated effect bordering on statistical significance would necessarily become significant with increased sample size. In multi-pollutant models, power calculations can reflect the power for estimating the effect of each pollutant given a specified correlation with other pollutants, but such calculations do not address the adequacy of the control for confounding or differential measurement error, which can be major issues in such models [5,24]. Similarly, in any model, adequate statistical power does not ensure validity of model results, as there still may be problems compromising validity (e.g. lack of control for confounding, misspecification of dose response functions, measurement error). The validity of power analyses is also contingent on analyses being conducted in the proper framework. One can spuriously increase the probability of finding significant effects by using procedures such as data mining. Finally, the accuracy of power estimates from simulations based on data from a short time period depends on the degree to which the short time period is representative of the period in the planned study with respect to daily counts, the magnitude of associations between air pollution and outcomes, and the relationships between the various variables in the model.

## Conclusions

The findings of these simulations have several implications for the design of studies of acute air pollution health effects. Such studies often model many pollutant-outcome combinations, and power will be better for some hypotheses than for others. The issue of sufficient power should be viewed as a continuum and not a dichotomous (yes or no) issue. To optimize power, one can increase either time-series length (by acquiring data for a longer time period) or the daily outcome counts (e.g., by acquiring data from more hospitals). While mathematically both have a similar impact on power, the actual impact might not be the same if increasing time series length changes pollutant distributions and the relationships between pollutants and other variables in the model. Allowance should also be made for the impact on power of controlling for covarying pollutants. Finally, power estimates obtained from standard software were very close to those from simulations, but care is needed in selecting proper values for the software input parameters.

## Abbreviations

PM_2.5_: Particulate matter less than 2.5 microns in diameter; CO: Carbon monoxide; EC: Elemental carbon; CVD: Cardiovascular disease outcome group; CA: Cardiac arrest; RR: Risk ratio; μg/m³: Micrograms per cubic meter; ppmV: Parts per million by volume; °C: Degrees Celsius; ED: Emergency department; ICD-9: International Classification of Diseases, 9^th^ revision.

## Competing interests

The authors declare that they have no competing interests.

## Authors’ contributions

MK conceptualized the study, designed the simulations, ran initial simulations for scenarios, assisted with power calculations using statistical software, interpreted the results, and critically reviewed the manuscript. AW ran simulations to expand some of the scenarios, performed power calculations using statistical software, participated in interpretation of results, and prepared the manuscript. SS and PT participated in conceptualizing the paper and interpretation of the results, and critically reviewed the manuscript. All authors read and approved the final manuscript.
